# Dual “Bacterial-Fungal” Symbiosis in Deltocephalinae Leafhoppers (Insecta, Hemiptera, Cicadomorpha: Cicadellidae)

**DOI:** 10.1007/s00248-017-1075-y

**Published:** 2017-09-23

**Authors:** Michał Kobiałka, Anna Michalik, Marcin Walczak, Teresa Szklarzewicz

**Affiliations:** 1 Department of Developmental Biology and Morphology of Invertebrates, Institute of Zoology and Biomedical Research, Jagiellonian University, Gronostajowa 9, 30-387 Kraków, Poland; 2 Department of Zoology, Faculty of Biology and Environmental Protection, University of Silesia, Bankowa 9, 40-007 Katowice, Poland

**Keywords:** Leafhoppers, Yeast-like microorganisms, Symbionts, *Ophiocordyceps*, *Sulcia*, Transovarial transmission

## Abstract

The symbiotic systems (types of symbionts, their distribution in the host insect body, and their transovarial transmission between generations) of four Deltocephalinae leafhoppers: *Fieberiella septentrionalis*, *Graphocraerus ventralis*, *Orientus ishidae*, and *Cicadula quadrinotata* have been examined by means of histological, ultrastructural, and molecular techniques. In all four species, two types of symbionts are present: bacterium *Sulcia* (phylum Bacteroidetes) and yeast-like symbionts closely related to the entomopathogenic fungi (phylum Ascomycota, class Sordariomycetes). *Sulcia* bacteria are always harbored in giant bacteriocytes, which are grouped into large organs termed “bacteriomes.” In *F. septentrionalis*, *G. ventralis*, and *O. ishidae*, numerous yeast-like microorganisms are localized in cells of the fat body, whereas in *C. quadrinotata*, they occupy the cells of midgut epithelium in large number. Additionally, in *C. quadrinotata*, a small amount of yeast-like microorganisms occurs intracellularly in the fat body cells and, extracellularly, in the hemolymph. *Sulcia* bacteria in *F. septentrionalis*, *G. ventralis*, *O. ishidae*, and *C. quadrinotata*, and the yeast-like symbionts residing in the fat body of *F. septentrionalis*, *G. ventralis*, and *O. ishidae* are transovarially transmitted; i.e., they infect the ovarioles which constitute the ovaries.

## Introduction

The symbiotic microorganisms living in the body of some insects have a large impact on their development, growth, and survival and, consequently, on their evolution [1–3]. Hemiptera: Auchenorrhyncha (Fulgoromorpha (planthoppers) and Cicadomorpha (leafhoppers, treehoppers, spittlebugs, and cicadas)) are known for their great diversity of symbiotic systems (i.e., types of symbionts, their distribution in the body of the host insect, and the mode of their transmission from generation to generation) [4–10]. Since the plant sap consumed by these hemipterans contains an insufficient amount of amino acids necessary for their proper functioning, the ancestors of extant phloem and xylem feeders acquired microorganisms, which are a source of essential substances missing in their diet [11–13]. As a result of an ancient infection, the symbiotic microorganisms are present in all the members of the particular taxa of insects. The symbionts of auchenorrhynchans are harbored in the specialized organs of the host insect termed bacteriomes or mycetomes. Microbial mutualists are passed from mother to offspring transovarially [14]. Another characteristic feature of bacterial symbionts is their highly reduced genome, which is the consequence of a very long co-evolution between the microorganism and its host insect [2, 6, 15].

The histological studies of Müller [16] and Buchner [14] as well as later ultrastructural and molecular analyses [4–7, 10, 17–21] have shown that auchenorrhynchans are, as a rule, colonized by at least two obligate symbiotic microorganisms. As all these symbionts provide essential amino acids to the host, they have been named “co-primary symbionts” [4]. Molecular analyses of both symbionts and host insects have shown that the ancient symbiont of Auchenorrhyncha was a member of the Bacteroidetes—bacterium “*Candidatus* Sulcia muelleri” (hereafter *Sulcia*), which infected the auchenorrhynchan’s ancestor over 260 million years ago [22]. In ancestral auchenorrhynchans, *Sulcia* co-resided with one additional symbiont, which was a member of the class Betaproteobacteria. Most auchenorrhynchans retained the ancestral betaproteobacterial symbionts; e.g., in Deltocephalinae leafhoppers, *Sulcia* co-occurs with “*Candidatus* Nasuia deltocephalinicola” (hereafter *Nasuia*) [6, 9, 17, 19, 23]; in froghoppers, with “*Candidatus* Zinderia insecticola” [7, 24]; and in planthoppers, with “*Candidatus* Vidania fulgoroidea” [5]. During evolution in some lineages, the ancestral betaproteobacterium was replaced by another symbiont—bacterium or yeast-like microorganism; e.g., most sharpshooters have two nutrient providers: *Sulcia* and gammaproteobacterium “*Candidatus* Baumannia cicadellinicola” (hereafter *Baumannia*) [4, 25] and cicadas—*Sulcia* and alphaproteobacterium “*Candidatus* Hodgkinia cicadicola” [26]. In some auchenorrhynchans, apart from the bacterium *Sulcia* and its co-symbiont, a third additional associate occurs, e.g., gammaproteobacterium *Arsenophonus* in *Macrosteles laevis* (Cicadellidae, Deltocephalinae) [9] and gammaproteobacterium *Sodalis* in *Aphrophora quadrinotata* (Cercopidae) [7]. Moreover, it was observed that in the green leafhopper *Cicadella viridis*, the novel bacterium *Baumannia* has been more recently replaced by the bacterium *Sodalis* [18]. In the eared leafhopper *Ledropsis discolor* (Cicadellidae, Ledrinae), the bacterium *Sulcia* is accompanied by yeast-like symbionts, whereas in *Ledra auditura* and *Tituria angulata* (both Cicadellidae: Ledrinae) [10], in leafhopper *Scaphoideus titanus* (Cicadellidae: Deltocephalinae) [27], and in some Delphacidae planthoppers examined so far (e.g., *Nilaparvata lugens*, *Sogatella furcifera*, *Laodelphax striatellus*) [28], ancestral bacterial symbionts have been eliminated and replaced by yeast-like symbionts. The above data demonstrate continuous and independent symbiont replacing throughout the evolution of the hemipteran lineages mentioned.

In this study, we describe the symbiotic system of four leafhoppers from the subfamily Deltocephalinae: *Fieberiella septentrionalis* (tribe Fieberiellini), *Graphocraerus ventralis* (tribe Athysanini), *Orientus ishidae* (tribe Athysanini), and *Cicadula quadrinotata* (tribe Cicadulini). The subfamily Deltocephalinae with over 6600 species distributed worldwide, classified into 38 tribes, is the biggest one within the Cicadellidae family [29]. The phylogeny and classification of Deltocephalinae leafhoppers are still a subject under discussion [29]. As results of earlier studies have indicated that members of the subfamily Deltocephalinae are characterized by very diverse symbiotic systems [6, 9, 17, 19, 21, 27, 30], we expect that our study will provide further details on the ultrastructure, distribution, systematic affiliation, and mode of transmission between generations of their symbiotic associates. While *F. septentrionalis*, *G. ventralis*, and *C. quadrinotata* are common in Poland, *O. ishidae* is a species native to Southeast Asia and adventive in Europe [31].

## Material and Methods

### Insects

Adult individuals (females) of *Fieberiella septentrionalis* (Wagner), *Graphocraerus ventralis* (Fallén), *Orientus ishidae* (Matsumura), and *Cicadula quadrinotata* (Fabricius) were collected during the late spring and summer, from April to September in the years 2014, 2015, and 2016 in the Polish cities of Kraków, Częstochowa, Katowice, and Bielsko-Biała. *F. septentrionalis* was collected from white swallow-wort *Vincetoxicum hirundinaria* (Apocynaceae). *F. septentrionalis*, as a pest of fruit trees and ornamental plants of Rosaceae family, is a species of economic significance [32]. *G. ventralis* was collected from *Poa pratensis* and *Anthoxanthum odoratum* (Poaceae) grasses. So far, there is no data on the economic/phytosanitary significance of *G. ventralis*. *O. ishidae* was collected from the midland hawthorn, *Crataegus oxyacantha* (Rosaceae). *O. ishidae* is a species of Asian origin which was introduced into Europe and is known as a vector of phytoplasma pathogens, which cause the flavescence dorée (FD) disease in grapevines [33] and peach X disease [34]. *C. quadrinotata* was collected from sedges, *Carex* spp. (Cyperaceae). To date, *C. quadrinotata* was not examined for the presence of plant pathogens.

### Light and Electron Microscopy

The abdomens of about 25 females of each examined species were fixed in 2.5% glutaraldehyde solution in 0.1 M phosphate buffer (pH 7.4) at 4 °C for 3 months. The samples were then rinsed using 0.1 M phosphate buffer with the addition of 5.8% sucrose and, after that, postfixed in 1% solution of osmium tetroxide in the same phosphate buffer. The material was dehydrated in a series of solutions of ethanol with an increased concentration and acetone and, finally, embedded in epoxy resin Epon 812 (SERVA, Heidelberg, Germany). The Epon blocks were cut into serial, semithin (1-μm-thick), and ultrathin (90-nm-thick) sections. The sections, stained in 1% methylene blue in 1% borax (for histological studies) or contrasted with lead citrate and uranyl acetate (for ultrastructural studies), were observed and photographed under a suitable microscope: the Nikon Eclipse 80i light microscope (LM) and JEOL JEM-2100 electron transmission microscope (TEM).

### DNA Analyses

The total genomic DNA was isolated from ten adult females of *O. ishidae*, *F. septentrionalis*, *G. ventralis*, and *C. quadrinotata*, previously fixed in 100% ethanol. The DNA was extracted using the Sherlock AX DNA and Genomic Mini AX Yeast extraction kits (A&A Biotechnology) following the manufacturer’s protocol and then stored at − 20 °C for further analyses.

The fungal 18S ribosomal DNA (rDNA) was amplified by a PCR performed with primers NS1 (5′-GTA GTC ATA TGC TTG TCT C-3′) [35] and FS2 (5′-TAG GNA TTC CTC GTT GAA GA-3′) [36] under the following conditions: an initial denaturation step at 94 °C for 3 min, followed by 33 cycles at 94 °C for 30 s, 54 °C for 40 s, and 70 °C for 1 min and 40 s and a final extension step of 5 min at 72 °C. The PCR product was made visible by the use of electrophoresis in 1.5% agarose gel stained with Midori Green (Nippon Genetics Europe), and next, the appropriate bands were cut and purified using the Gel-out purification kit (A&A Biotechnology). The purified PCR product was cloned to the pJET1.2/blunt plasmid vector using the CloneJET PCR Cloning Kit (Thermo Scientific). The ligated mixtures were then transformed into competent *Escherichia coli* TOP10F cells which were prepared using the *E. coli* Transformer Kit (A&A Biotechnology). After 16 h, the occurrence of the fungal 18S rDNA was confirmed by diagnostic PCRs from colonies with the following primers: pJET For. (5′-GCCTGAACACCATATCCATCC-3′) and pJET Rev. (5′-GCAGCTGAGAATATTGTAGGAGAT-3′). Thirty positive colonies of each analyzed species were subjected to restrictive analysis using an *Msp*I restriction enzyme. The plasmids from the selected colonies were isolated using a Plasmid Mini AX kit (A&A Biotechnology) and then sequenced. The Sanger sequencing reactions were performed using the BigDye^®^ Terminator v3.1 kit (Life Technologies). For each sequencing reaction, 3 μl BigDye™ Terminator v3.1 Ready Reaction Mix, 1 μl BigDye™ Terminator v1.1 and v3.1 5× sequencing buffer, 5 pmol of the appropriate primer, and 50–250 ng of DNA template were finally mixed in a 10 μl volume. Cycle sequencing was performed in 100-μl PCR tubes. Incubation took place at 96 °C for 1 min as initial denaturation step, followed by 25 cycles of 96 °C for 10 s, 54 °C for 5 s, and 60 °C for 4-min incubation. In the prior purification, reaction mixture was then incubated at 4 °C. The purified reaction products were separated by electrophoresis on the 3730xl DNA Analyzer, following the manufacturer’s references (Thermo Fisher). Molecular cloning was performed for two individuals of each of the species examined.

The 18S rDNA sequence of yeast-like symbiont of *G. ventralis* was not obtained in the PCR using primer NS1/FS2 despite the fact that the presence of these symbionts was confirmed by histological and ultrastructural analyses. A similar situation was described by Nishino and co-workers [10], who examined yeast-like symbionts of the other leafhopper—*Ledropsis discolor*. For this reason, in order to establish the systematic affinity of the yeast-like symbionts of *G. ventralis*, the 28S rDNA sequence was amplified using primers SymbioT.FWD (5′-AGG GAT TGC CTC AGT AAC GG-3′) and SymbioT.REV (5′-GAC ACC CAA ACA CTC GCA TA-3′) designed using Primer3 software based on available sequences deposited in the GenBank database (Vanderpool, unpublished).

The 16S rDNA genes of *Sulcia* symbionts of the examined species of Deltocephalinae were amplified in PCR using *Sulcia*-specific primers 10CFBF (5′-AGAGTTTGAATCATGGCTCAGGATG-3′) and 1515R (5′-GTACGGCTACCTTGTTACGACTTAG-3′) [22] under the above conditions. The product of the PCRs was checked for specificity in 1.5% agarose electrophoresis gel stained with Midori Green (Nippon Genetics Europe), and after that, the samples were subjected to sequencing. The nucleotide sequences obtained were deposited in the GenBank database under the accession numbers MF536295 and KY923021–KY923029.

### Phylogenetic Analysis

The phylogenetic analysis of the *Sulcia* symbionts was performed on the basis of the sequences of their 16S rDNA, whereas for phylogenetic analysis of yeast-like symbionts, their 18S rDNA sequences were used. First, the sequences were edited using BioEdit Sequence Alignment Editor 5.0.9 [37], and the alignments were generated using Clustal X 1.8 [38]. The phylogenetic analyses were conducted using MrBayes 3.2.2 (Bayesian analysis) and MEGA7.0 (maximum likelihood analysis) software [39, 40]. In the Bayesian analyses, four incrementally Metropolis-coupled MCMC chains (three heated and one cold) were run for ten million generations. The results of the Bayesian analyses were put into visual form using FigTree 1.4.0 software [41].

## Results

### Ultrastructure and Distribution of Symbiotic Microorganisms

**Fig. 1:**
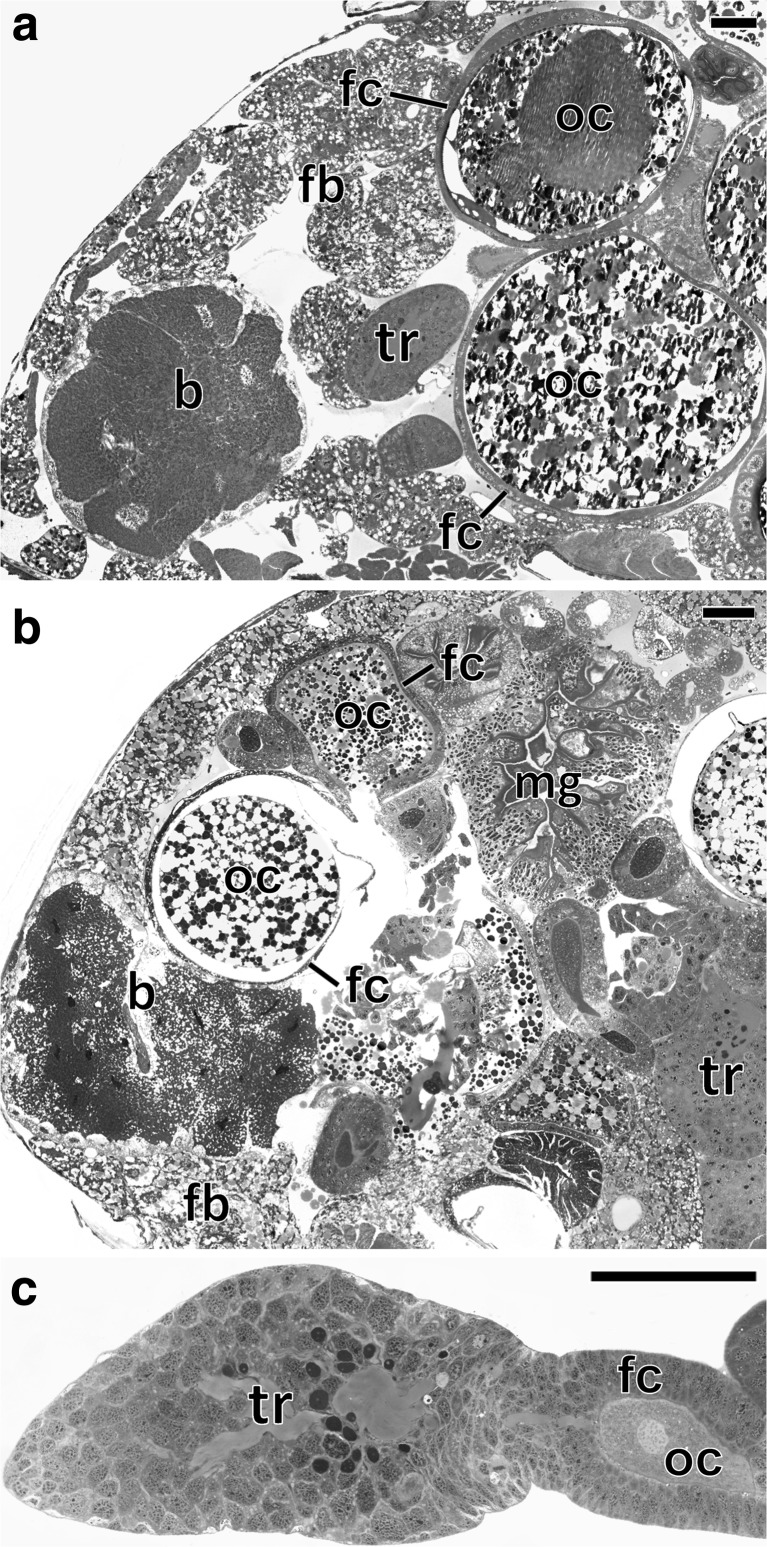
Localization of bacteriomes and ovaries in the abdomen of an adult female. **a** Fragment of the abdomen of *Graphocraerus ventralis* (cross section). **b** Fragment of the abdomen of *Cicadula quadrinotata* (cross section). **c** Fragment of the anterior region of the ovariole of *Orientus ishidae* (longitudinal section). **a**–**c** LM, methylene blue, scale bar = 25 μm; b bacteriome with bacterium *Sulcia*; fb fat body lobe; fc follicular epithelium; oc oocyte; mg midgut; tr tropharium

**Fig. 2:**
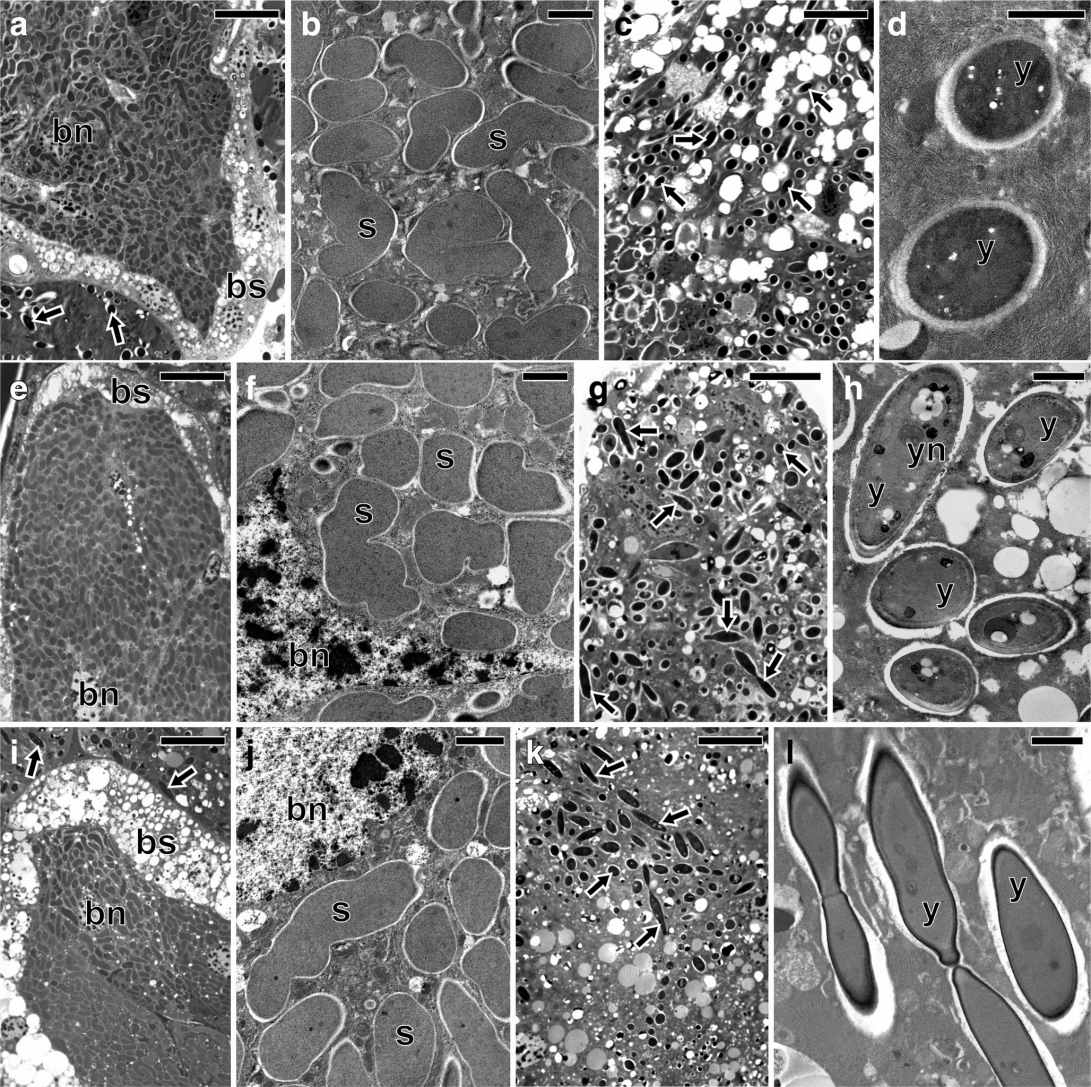
Distribution of bacterial and yeast-like symbionts in the body of *Fieberiella septentrionalis* (**a**–**d**), *Graphocraerus ventralis* (**e**–**h**), and *Orientus ishidae* (**i**–**l**). **a**, **b**, **e**, **f**, **i**, **j** Fragment of the bacteriome with bacterium *Sulcia*. **c**, **d**, **g**, **h**, **k**, **l** Fragment of the fat body lobe with yeast-like microorganisms. **a**, **c**, **e**, **g**, **i**, **k** LM, methylene blue, scale bar = 25 μm. **b**, **d**, **f**, **h**, **j**, **l** TEM, scale bar = 2 μm. Black arrows (in LM images) and y (in TEM images) yeast-like microorganisms in the fat body cells; bn bacteriocyte nucleus; bs bacteriome sheath; s *Sulcia*; yn nucleus of the yeast-like microorganism

**Fig. 3:**
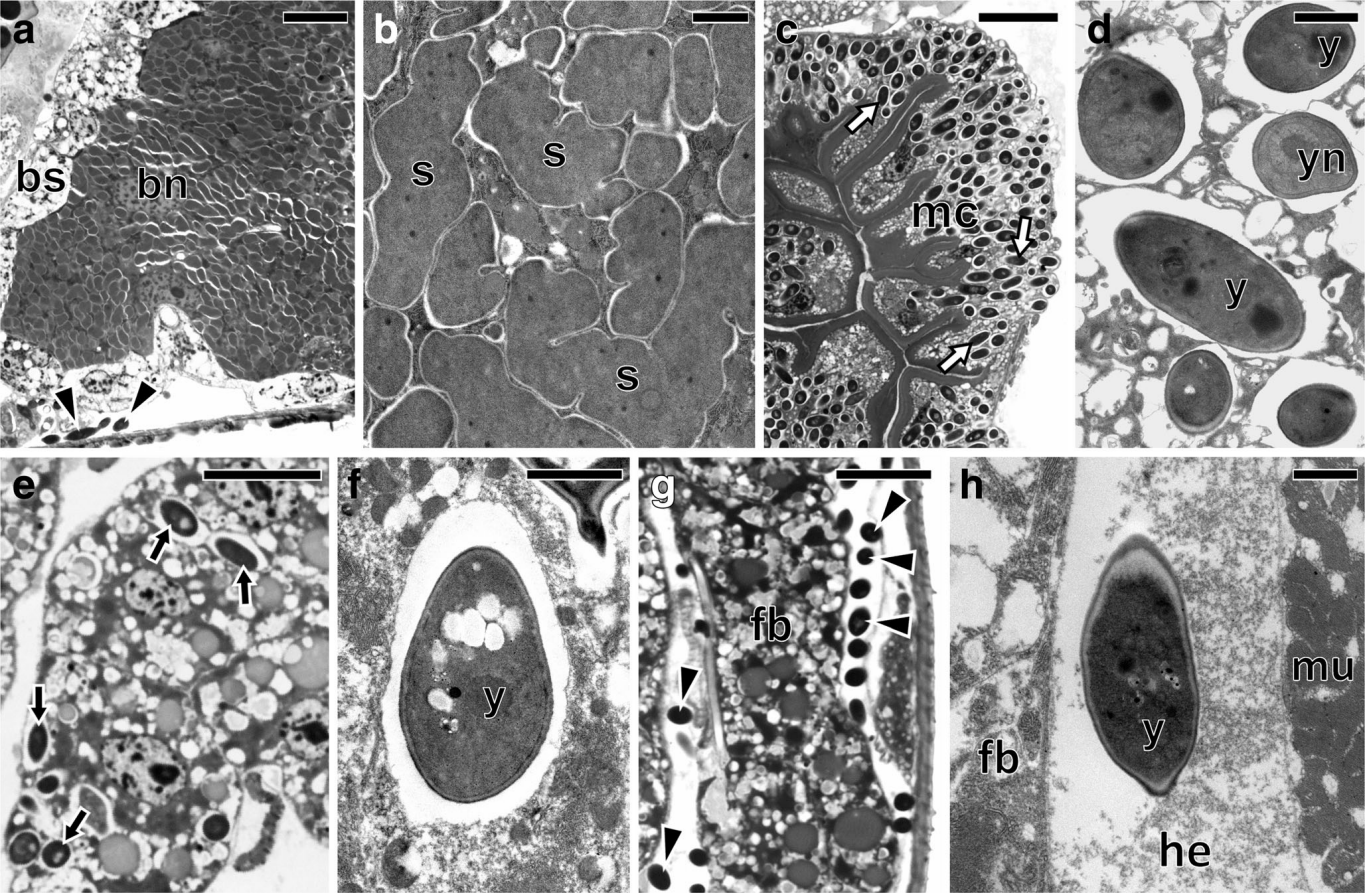
Distribution of bacterial and yeast-like symbionts in the body of *Cicadula quadrinotata*. **a**, **b** Fragment of the bacteriome with bacterium *Sulcia*. **c**, **d** Fragment of the midgut with yeast-like microorganisms in the cells of the midgut epithelium. **e**, **f** Fragment of the fat body lobe with intracellularly localized yeast-like microorganisms. **g**, **h** Yeast-like microorganisms in the hemolymph. **a**, **c**, **e**, **g** LM, methylene blue, scale bar = 25 μm. **b**, **d**, **f**, **h** TEM, scale bar = 2 μm. White arrows yeast-like microorganisms in the midgut cells; black arrows yeast-like microorganisms in the fat body cells; black arrowheads yeast-like microorganisms in the hemolymph; bn bacteriocyte nucleus; bs bacteriome sheath; fb fat body lobe; he hemolymph; mc cells of midgut epithelium; mu muscles; s *Sulcia*; y yeast-like microorganism; yn nucleus of the yeast-like microorganism

The ultrastructural and histological analyses revealed the presence of two large bacteriomes localized ventro-laterally, on both sides of the abdomen of each studied species: *Fieberiella septentrionalis*, *Graphocraerus ventralis*, *Orientus ishidae*, and *Cicadula quadrinotata*. These organs are located between the body wall and the gonads (Fig. 1a, b) and are surrounded by a thin monolayered epithelium called the bacteriome sheath (Figs. 2a, e, i and 3a). Ultrastructural observations did not reveal symbiotic microorganisms in the cells of the bacteriome sheath. Bacteriomes are composed of giant bacteriocytes, which have large, irregular, branched nucleus and cytoplasm tightly packed with pleomorphic bacteria (Figs. 2a, b, e, f, i, j and 3a, b). In the fat body cells of the individuals of the three species studied: *F. septentrionalis*, *G. ventralis*, and *O. ishidae*, numerous yeast-like symbionts have been observed (Fig. 2c, d, g, h, k, l). It was observed that in the cells of the fat body of *C. quadrinotata* (Fig. 3e), yeast-like symbionts are far fewer than in *F. septentrionalis*, *G. ventralis*, and *O. ishidae*. A few yeast-like microorganisms were also observed in the hemolymph of *C. quadrinotata* (Fig. 3a, g). A large amount of yeast-like microorganisms has been found inside cells of the midgut epithelium of *C. quadrinotata* (Fig. 3c, d). Fungal microorganisms living intracellularly in fat body cells and in cells of midgut epithelium, as well as extracellularly in the hemolymph have a characteristic, elongated shape (Figs. 2h, l and 3d, f, h) and measure about 3–3.5 μm in diameter. The cells of the yeast-like symbionts are surrounded by a thick cell wall (Figs. 2h and 3d, f, h). They possess a large, spherical nucleus with a single electron-dense nucleolus (Figs. 2h and 3d).

### Molecular Identification of Microorganisms

**Fig. 4:**
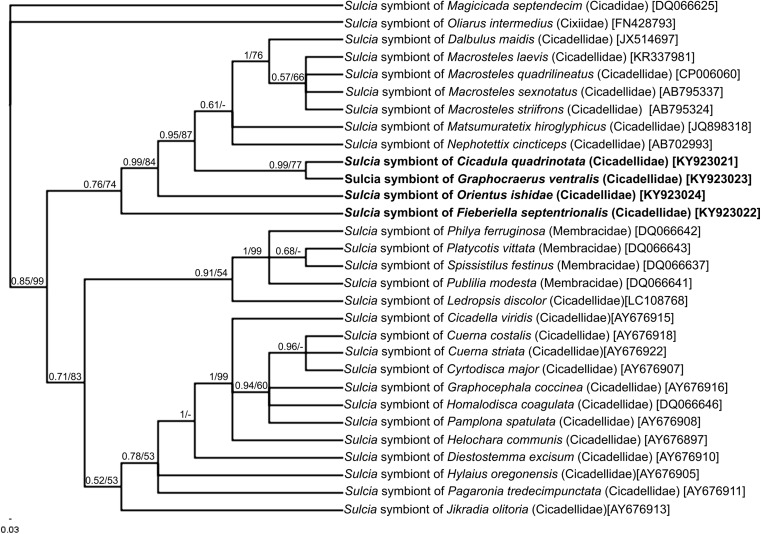
Bayesian cladogram showing the relationships of *Sulcia* symbionts of the examined leafhoppers and other representatives of Cicadellidae and Membracidae families. The phylogenetic analysis was conducted on the basis of 16S rRNA gene sequences. The numbers associated with the branches indicate the Bayesian posterior probabilities and maximum likelihood bootstrap values, respectively. The accession numbers of the sequences used in the phylogenetic analysis have been placed in brackets. For outgroups, *Sulcia* symbionts of *Magicicada septendecim* (Cicadidae) and *Oliarus intermedius* (Fulgoromorpha: Cixiidae) were used

Histological and ultrastructural observations have shown that all the leafhoppers of the Deltocephalinae subfamily examined are hosts to both prokaryotic and eukaryotic microorganisms. Due to the fact that we have observed only one type of bacterial symbiont that was similar in shape, size, and ultrastructure to the *Sulcia* bacteria—previously observed in other genera of Deltocephalinae [9, 19, 21, 23], we used *Sulcia*-specific primers for the detection of the presence of these symbionts. The comparison of the sequences obtained with the homologous sequences deposited in the GenBank database using BLAST has confirmed that the bacteria residing in the bacteriocytes of examined species of Deltocephalinae belong to the genus *Sulcia*. The 16S rDNA sequence of *Sulcia* symbiont of *F. septentrionalis*, *G. ventralis*, *O. ishidae*, and *C. quadrinotata* displays a high similarity to homologous *Sulcia* sequence isolated from *G. ventralis* (97% similarity), *C. quadrinotata* (99% similarity), *Ecultanus excultus* (97% similarity), and *Nephotettix cincticeps* (99% similarity). The phylogenetic analysis based on 16S rDNA sequences has shown that *Sulcia* symbionts of Deltocephalinae form a monophyletic group with moderate support (Bayesian posterior probability = 0.76, bootstrap support = 74%) (Fig. 4). Both methods used for the phylogenetic analysis (Bayesian and maximum likelihood methods) confirmed the close relationships between 16S rDNA sequences of *Sulcia* symbionts of Deltocephalinae leafhoppers.

**Fig. 5:**
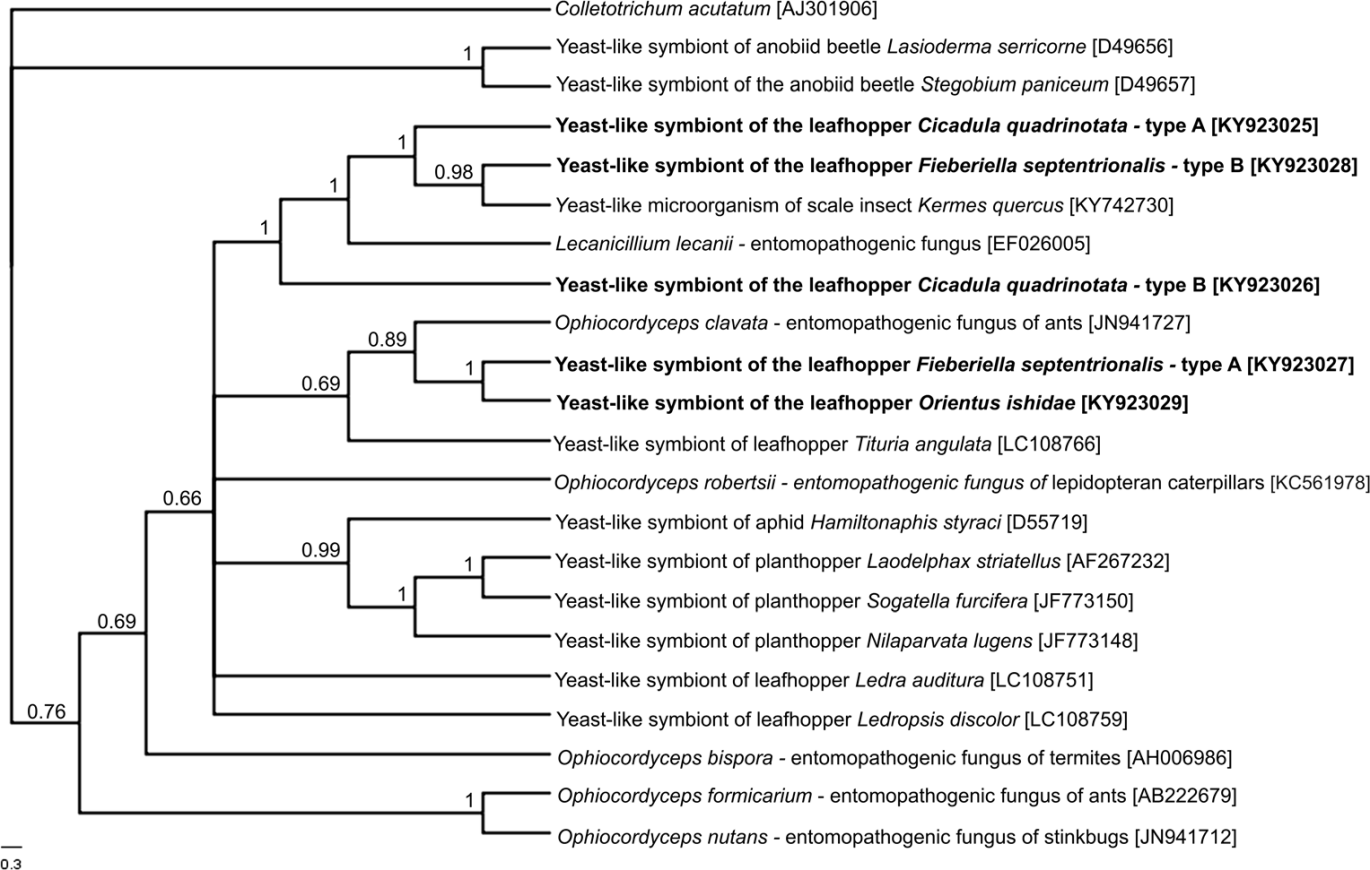
Bayesian cladogram showing the phylogenetic placement of the yeast-like microorganisms of examined leafhoppers. The phylogenetic analysis was conducted based on 18S rRNA gene sequences. The numbers associated with the branches indicate the Bayesian posterior probabilities. The accession numbers of the sequences used in the phylogenetic analysis have been placed in brackets. For outgroup, the pathogenic fungus *Colletorichum acutatum* was used

For the identification of eukaryotic microorganisms, we have used the universal fungal primers for 18S rDNA recommended by White et al. [35] and Nikoh and Fukatsu [36]. In order to determine the diversity of the fungal microorganisms of the examined species, the PCR products were subjected to molecular cloning. The results of the RFLP analyses using the *Msp*I restriction enzyme have shown that all the individuals of *O. ishidae* are host to only one type of fungal symbiont, whereas all the specimens of *F. septentrionalis* and *C. quadrinotata* harbor two types of eukaryotic microorganisms. The Bayesian analysis has revealed that both types of yeast-like symbionts of *C. quadrinotata* (designated as types A and B) as well as one type of the yeast-like symbiont of *F. septentrionalis* (designated as type B) constitute a well-supported cluster (1.00 posterior probability) with the entomopathogenic fungus *Lecanicillium lecanii* (an anamorphic form of the genus *Cordyceps*) and yeast-like microorganisms which reside in the fat body cells in the scale insect *Kermes quercus* (Fig. 5). Yeast-like symbionts detected in *O. ishidae* and the second type of yeast-like microorganisms of *F. septentrionalis* (designated as type A) form a clade with the yeast-like symbiont of the leafhopper *Tituria angulata* and *Ophiocordyceps clavata*—the entomopathogenic fungus of ants (Fig. 5).

As stated in the “Material and Methods” section, for the identification of the yeast-like symbionts of *G. ventralis*, the 28S rDNA sequence (759 bp, accession number MF536295) was used. BLAST searches have confirmed that fungal symbionts of *G. ventralis* also are closely related to an entomopathogenic fungus *Ophiocordyceps* and display the highest similarity (91%) to the 28S rDNA sequence of fungus *Ophiocordyceps coccidicola*, isolated from scale insects.

### Transovarial Transmission of Symbiotic Microorganisms

**Fig. 6:**
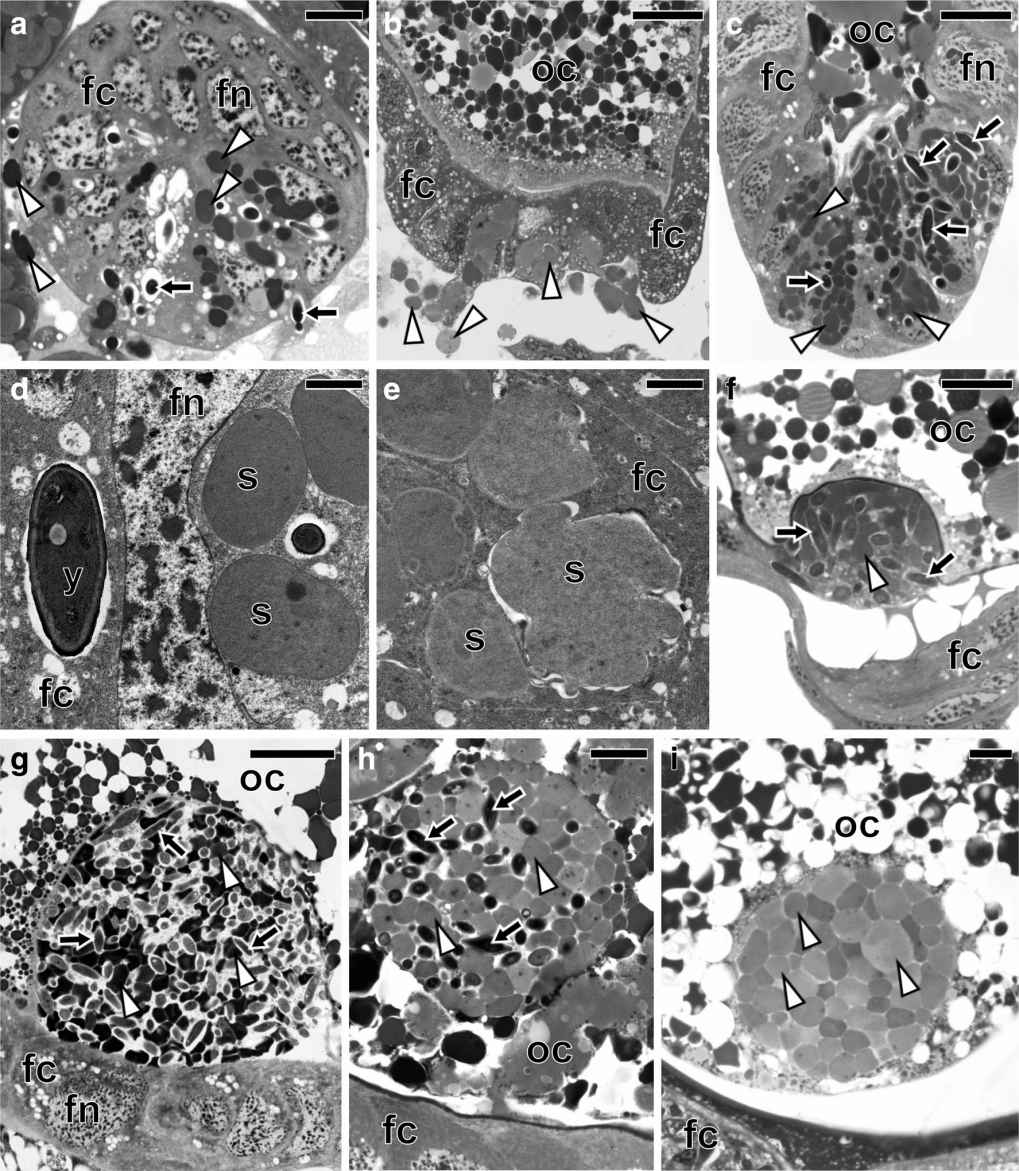
Consecutive stages of the infestation of ovaries by symbionts in *Fieberiella septentrionalis*, *Graphocraerus ventralis*, *Orientus ishidae*, and *Cicadula quadrinotata*. **a** *G. ventralis*. *Sulcia* bacteria and yeast-like microorganisms start to invade follicular cells (cross section). **b** *C. quadrinotata*. The ovariole is infected by *Sulcia* bacteria only (longitudinal section). **c** *F. septentrionalis*. *Sulcia* bacteria and yeast-like microorganisms migrate through the cytoplasm of follicular cells (longitudinal section). **d** *G. ventralis*. Yeast-like microorganism and *Sulcia* bacteria in the cytoplasm of follicular cell. **e** *C. quadrinotata*. *Sulcia* bacteria in the cytoplasm of follicular cell. **f** *O. ishidae*. *Sulcia* bacteria and yeast-like microorganisms begin to gather in the perivitelline space (longitudinal section). **g**, **h** *F. septentrionalis* and *G. ventralis*, respectively. A *symbiont ball* composed of *Sulcia* bacteria and yeast-like microorganisms. **i** *C. quadrinotata*. A symbiont ball containing only *Sulcia* bacteria. **a**–**c**, **f**–**i** LM, methylene blue, scale bar = 25 μm. **d**, **e** TEM, scale bar = 2 μm. Black arrows (in LM images) and y (in TEM images) yeast-like microorganisms; white arrowheads (in LM images) and s (in TEM images) bacterium *Sulcia*; fc follicular cells; fn nucleus of follicular cell; oc oocyte

Observations of the ovaries of reproductive females (i.e., containing ovaries with vitellogenic oocytes) (Fig. 1a, b) have revealed that in *F. septentrionalis*, *G. ventralis*, and *O. ishidae*, both the symbiotic bacteria and yeast-like symbionts are transovarially transmitted from mother to offspring, whereas in *C. quadrinotata*, our observations indicate that only bacterium *Sulcia* appears to be transovarially inherited. In adult females of the species studied, the ovaries consist of several ovarioles of a telotrophic-meroistic type (Fig. 1c) (for the classification and organization of insect ovarioles, see Büning [42] and Biliński [43]). Each ovariole contains several linearly arranged oocytes, which are surrounded by a single layer of follicular cells (Figs. 1a–c and 6b, c, g). At the same time as the *Sulcia* bacteria leave the cytoplasm of bacteriocytes, the yeast-like symbionts leave the cytoplasm of the fat body cells and then both of these microorganisms begin to invade the ovarioles. In *C. quadrinotata*, the ovarioles are only infested by the bacterium *Sulcia* (Fig. 6b). The symbionts migrate to the terminal oocytes in the stage of advanced vitellogenesis and gather around their posterior poles (Fig. 6a, b). During migration, *Sulcia* bacteria change their shape from pleomorphic into a more ovoid or even almost spherical (Fig. 6a, b). Next, the symbionts enter the cytoplasm of the follicular cells (Fig. 6a–e). As numerous symbiotic microorganisms accumulate in the cytoplasm of the follicular cells, their volume increases greatly (Fig. 6c). After leaving the follicular cells, the microorganisms accumulate in the space between the oocyte and follicular epithelium, called the perivitelline space (Fig. 6f). The symbionts then assemble in the deep invagination of the oolemma and closely adhere to one another to form a tightly packed structure termed a “symbiont ball” (Fig. 6g–i). In *F. septentrionalis*, *G. ventralis*, and *O. ishidae*, the symbiont ball contains both bacterial and fungal symbionts (Fig. 6f–h), whereas in *C. quadrinotata*, only bacterial symbionts are present (Fig. 6i). As in the case of other auchenorrhynchans so far examined [9, 18–20], the symbionts do not enter the ooplasm until the end of oogenesis.

## Discussion

Our observations have revealed that the Deltocephalinae leafhoppers examined: *Fieberiella septentrionalis*, *Graphocraerus ventralis*, *Cicadula quadrinotata*, and *Orientus ishidae* are host to the ancient symbiont—bacterium *Sulcia* and yeast-like symbionts. Thus, these findings provide further arguments supporting the view that the subfamily Deltocephalinae is characterized by diverse symbiotic systems. Previous studies on symbionts in Deltocephalinae leafhoppers have shown that some of them (e.g., *Matsumuratettix hiroglyphicus*, *Nephotettix cincticeps*, *Macrosteles quadrilineatus*, *Deltocephalus pulicaris*) retained the ancient dual symbiotic system consisting of the bacterium *Sulcia* and the betaproteobacterium *Nasuia* [6, 19, 23, 44]. In *Macrosteles laevis* aside from these two ancient symbionts, the novel symbiont, i.e., the gammaproteobacterium *Arsenophonus*, common among arthropods, is present [9]. What is of special interest is that the *Arsenophonus* bacteria have not been observed individually in *M. laevis* but were always found internalized in the cells of *Sulcia* bacteria. It should furthermore be stressed that a similar phenomenon of nested symbiosis has been reported in Pseudococcidae mealybugs [45–51], as well as in the leafhopper *Cicadella viridis* [18]. Kobiałka and co-workers [9] suggest that the internalized *Arsenophonus* bacterium, similar to *Sodalis*-like bacterium in Pseudococcidae mealybugs [45] and in the leafhopper *C. viridis* [18], represents a newly acquired symbiont in *M. laevis*, which cannot yet be transmitted to offspring on its own. The co-occurrence in *M. laevis* of two ancient symbionts and the symbiont of a more recent origin indicates that the betaproteobacterial symbiont has not yet been eliminated, whereas the new symbiont has already been acquired. On the other hand, a lack of the ancient betaproteobacterium *Nasuia* in *S. titanus*, *F. septentrionalis*, *G. ventralis*, *C. quadrinotata*, and *O. ishidae* [27, this study] suggests that in some species of Deltocephalinae leafhoppers, this bacterium has been already eliminated and replaced by yeast-like symbionts. It should be stressed that a similar evolutionary scenario has been presented by Nishino and co-workers [10], who studied the symbiotic systems of three members of the Ledrinae leafhoppers. Similarly, a lack of *Nasuia* inside the bacteriomes of the Deltocephalinae *Dalbulus maidis* was reported by Brentassi and co-workers [21], who suggested that the leafhopper may have lost some of their symbiotic species during phylogeny.

The results of earlier studies using a paraffin technique [14, 16], and more recent ultrastructural and molecular analyses [this study], indicate that the yeast-like symbionts are rather uncommon in members of Deltocephalinae leafhoppers. The use of molecular phylogenetic analyses revealed that yeast-like symbionts residing in *F. septentrionalis*, *G. ventralis*, *C. quadrinotata*, and *O. ishidae* (see Fig. 5) are closely related to the fungi from the genera *Ophiocordyceps* and *Cordyceps*, which include a widely distributed fungal entomopathogens [52]. These findings thus indicate that the fungal entomopathogens, in contrast with the ancient symbiont, i.e., bacterium *Sulcia*, infected the ancestors of studied species independently from each other. Then, the acquired fungi evolved into mutualistic symbionts. Suh and co-workers [53], who studied the yeast-like symbionts of planthoppers, postulated that during co-evolution, the insect-fungus interaction changed, resulting in modifications in the morphology, life cycle, and physiology of fungal entomoparasites. These fungi lost their previous filamentous ascomycete form and remained only in a yeast-like form. It is worth mentioning that besides the alterations in the morphology of yeast-like symbionts residing in insects, changes within their genome also occurred. Recently, Fan and co-workers [54] have shown that during the co-evolution of yeast-like symbionts and its host insect, the brown planthopper *Nilaparvata lugens*, the loss of some genes of yeast-like microorganisms took place.

So far, the fungal symbionts related to entomopathogens have been found in aphids [55], in planthoppers from the Delphacidae family [28, 53], in anobiid beetles [56], in scale insects from the Kerriidae, Dactylopiidae, and Kermesidae families [57–59], and in leafhoppers from the Ledrinae subfamily [10]. In most of the above cases, the yeast-like microorganisms are the predominant symbionts which reside in their host insects.

In *L. discolor*, *S. titanus*, *F. septentrionalis*, *G. ventralis*, and *O. ishidae*, numerous yeast-like microorganisms are localized in cells of the fat body [10, 27, this study], whereas in *C. quadrinotata* [this study], they occur both in the cells of the midgut epithelium (in a large amount) and in the fat body cells and hemolymph (in a small amount). It should be stressed that Buchner [14] found yeast-like microorganisms in the cells of midgut epithelium of *C. quadrinotata* but did not observe these microorganisms in the fat body. There are two possible explanations for this discrepancy: (1) the yeast-like symbionts may be present in the fat body cells and hemolymph of only certain populations of *C. quadrinotata* and (2) on account of the paraffin technique used, Buchner might have overlooked these microorganisms. The function of yeast-like microorganisms residing in the fat body and hemolymph of *C. quadrinotata* remains unknown; however, as these microorganisms are present in all individuals of *C. quadrinotata* and do not have a negative effect on the growth and development of the host insects, it may be possible that they represent an additional, newly acquired symbiont. It can also not be ruled out that the latter microorganisms may be facultative symbionts residing in the examined population of *C. quadrinotata*. Thus, to elucidate the biological role of these microorganisms, further experiments are needed.

Yeast-like microorganisms and bacterial symbionts in Deltocephalinae leafhoppers: *F. septentrionalis*, *G. ventralis*, and *O. ishidae* [this study], as well as in *S. titanus* [27], are transmitted transovarially. It was observed that in all the above insects, the symbionts migrate from the fat body towards the ovaries; then, via the cytoplasm of the follicular cells surrounding the posterior pole of the terminal oocytes, they enter the space between the oocyte and follicular epithelium. It should be stressed that the same manner of transmission of both the bacterial and fungal symbionts has been observed in other auchenorrhynchans [9, 14, 19, 27, 60], which confirms the earlier observations that these hemipterans, in spite of a large diversity of symbionts, developed a uniform mode of symbiont transmission [9, 18–21]. In contrast to the mode of symbiont transmission mentioned above, the style of inheritance of yeast-like microorganisms in *C. quadrinotata* remains unknown. Our observations clearly indicate (see Fig. 6i) that in this species, only bacterial symbionts enter the ovaries. Thus, the yeast-like microorganisms must be transmitted via a different route. Buchner [14] hypothesized that the yeast-like microorganisms of *C. quadrinotata*, similarly to the gut bacteria in heteropterans and yeast-like symbionts in beetles, may contaminate the egg surface. Newly hatched larvae consuming symbionts become infected with them. The lack of a mechanism ensuring the transovarial transmission of yeast-like symbionts in *C. quadrinotata* indicates that these symbionts were more recently acquired than the bacterial symbionts and yeast-like symbionts of other Deltocephalinae leafhoppers.

The presence of numerous yeast-like symbionts in the fat body or midgut epithelium of the species studied suggests that these microorganisms have an important metabolic function to their hosts. Data in the literature indicate that yeast-like symbionts may play varying roles; e.g., they may be engaged in the detoxification of food compounds in various beetles, termites, and wood wasps [61] and in amino acid metabolism, sterol biosynthesis, and nitrogen recycling in planthoppers [54, 62–64]. Nishino and co-workers [10] hypothesized that the large size of the genome of yeast-like symbionts, in comparison with the small genome size of bacterial symbionts, indicates that these fungal symbionts may play a broader biological function to the host insect. Therefore, in order to examine the role of yeast-like microorganisms, further genomic studies in combination with insect rearing and symbiont manipulation are required.
